# Wearables for Telemonitoring in ATTR-Amyloidosis: Current Perspectives

**DOI:** 10.3390/s26103035

**Published:** 2026-05-11

**Authors:** Andreas Kind, Helena Pernice, Gina Barzen, Jan Gröschel, Aurelian Schumacher, Stefanie Werhahn, Paul Wetzel, Frank Edelmann, Gerhard Hindricks, Katrin Hahn, Sebastian Spethmann

**Affiliations:** 1 Deutsches Herzzentrum der Charité (DHZC), Department of Cardiology, Angiology and Intensive CareMedicine, Charité Virchow Klinikum, 13353 Berlin, Germany; stefanie.werhahn@dhzc-charite.de (S.W.); frank.edelmann@dhzc-charite.de (F.E.); 2 Charité—Universitätsmedizin Berlin Corporate Member of Freie Universität Berlin and Humboldt Universität zu Berlin, 10117 Berlin, Germany; helena.pernice@charite.de (H.P.); gina.barzen@dhzc-charite.de (G.B.); jan.groeschel@dhzc-charite.de (J.G.); aurelian-eroni.schumacher@charite.de (A.S.); gerhard.hindricks@dhzc-charite.de (G.H.); katrin.hahn@charite.de (K.H.); 3 DZHK (German Centre for Cardiovascular Research), Partner Site Berlin, 10785 Berlin, Germany; 4 Amyloidosis Center Charité Berlin (ACCB), Amyloidosis Center of Charité, Campus Charité Mitte, Campus Benjamin Franklin and Campus Virchow Klinikum, 10117 Berlin, Germany; paul-julius.wetzel@charite.de; 5 Department of Neurology, Charité University Medicine Berlin, 10117 Berlin, Germany; 6 Deutsches Herzzentrum der Charité (DHZC), Department of Cardiology, Angiology and Intensive Care Medicine, Charité Campus Mitte, 10117 Berlin, Germany; 7 Berlin Institute of Health at Charité, Charité University Medicine Berlin, 10178 Berlin, Germany

**Keywords:** transthyretin amyloidosis, wearables, sensors, telemonitoring, heart failure, neuropathy, atrial fibrillation, gait analysis

## Abstract

Wearable sensors enable continuous recording of electrocardiographic, photoplethysmographic, and inertial signals and have accelerated the development of digital biomarkers in cardiovascular medicine. Transthyretin amyloidosis (ATTR) is a progressive multisystem disease characterized by arrhythmia, conduction disturbances, hemodynamic impairment, autonomic dysfunction, and gait abnormalities, making it theoretically suitable for multimodal wearable monitoring. This review summarizes current knowledge on wearable applications in amyloidosis with ATTR serving as an illustrative case, evaluates the plausibility of extrapolating signal-based biomarkers from related cardiovascular and neurological cohorts, and outlines methodological and implementation challenges. ATTR-specific data remain limited to small observational studies, mainly on long-term rhythm monitoring and supervised functional assessment. More comprehensive findings support the extraction of metrics such as atrial fibrillation burden, activity patterns, gait variability, and heart rate variability. However, ATTR-related structural remodeling and high arrhythmia burden may distort conventional digital biomarkers, necessitating disease-specific preprocessing and prospective validation. Wearable monitoring in ATTR is technically feasible and biologically plausible but remains investigational. Before routine integration into care pathways can be recommended, standardized, phenotype-stratified studies are needed that link wearable-derived characteristics to assessed clinical outcomes.

## 1. Introduction

Over the past two decades, wearable sensor technologies have evolved from consumer fitness devices to clinically relevant platforms for continuous physiological monitoring [1,2,3]. In cardiovascular medicine, wearables now enable high-resolution capture of electrocardiogram (ECG) signals, photoplethysmographic (PPG) waveforms, pulse oximetry, inertial motion data, acoustic signatures, and behavioral patterns under real-world conditions. This technological maturation has driven the development of digital biomarkers, which represent quantifiable, sensor-derived characteristics reflecting underlying biological processes [4]. Large-scale investigations such as the Apple Heart Study have demonstrated the scalable detection of atrial fibrillation (AF), while more recent approaches, including the VAMP-HF pilot study, have explored acoustic features from the voice as indicators of heart failure (HF) decompensation [5,6]. Complementary findings from telemonitoring studies suggest that continuous remote monitoring not only documents disease status but can also influence outcomes [7]. Research on digital biomarkers has now expanded beyond rhythm detection to include activity atrophy, gait variability, autonomic indices, sleep disturbances, and multimodal signal integration [4].

Transthyretin amyloidosis (ATTR) is a progressive protein folding disorder characterized by the extracellular deposition of transthyretin fibrils in multiple organ systems. Amyloid formation results either from destabilizing mutations in hereditary ATTR (ATTRv) or from age-related tetramer instability in wild-type ATTR (ATTRwt). The resulting involvement of multiple organ systems includes restrictive cardiomyopathy (ATTR-CM), infiltration of the conduction system, autonomic dysfunction, and progressive peripheral polyneuropathy (ATTR-PNP) [8,9].

ATTR is particularly well suited for digital phenotyping because the disease biology develops gradually across multiple physiological domains. In ATTRwt, restrictive cardiomyopathy typically manifests as heart failure with preserved ejection fraction (HFpEF), AF, conduction disturbances, and chronotropic incompetence, which are detectable in cardiopulmonary exercise testing (CPET) [10]. In ATTRv, mutation-specific phenotypes (e.g., p.Val50Met) often include progressive sensorimotor neuropathy and autonomic impairments that occur either before or together with cardiac involvement [11,12]. These manifestations correspond to measurable disturbances in electrical stability, hemodynamics, autonomic regulation, and motor function.

Before obvious clinical deterioration becomes apparent, the progression of ATTR is often accompanied by subtle physiological changes such as increasing arrhythmia burden, decreasing physical activity, altered autonomic variability, emerging gait asymmetry, or early fluid retention [13,14,15]. Despite the availability of disease-modifying ATTR therapies, clinical monitoring remains largely episodic. Such transitional stages are difficult to detect during clinical visits but are theoretically accessible through continuous time series derived from wearable sensors in at-risk patients.

Unlike acute inflammatory or ischemic cardiomyopathies, ATTR progresses slowly with cumulative structural remodeling. This temporal profile creates a convergence between the biology of the disease and digital capabilities: wearable sensors can longitudinally capture electrical, mechanical, and behavioral signals that evolve in parallel with pathophysiological changes. However, to translate this potential into clinical utility, sensor-derived features must be explicitly mapped to disease-relevant biological domains.

**Figure 1:**
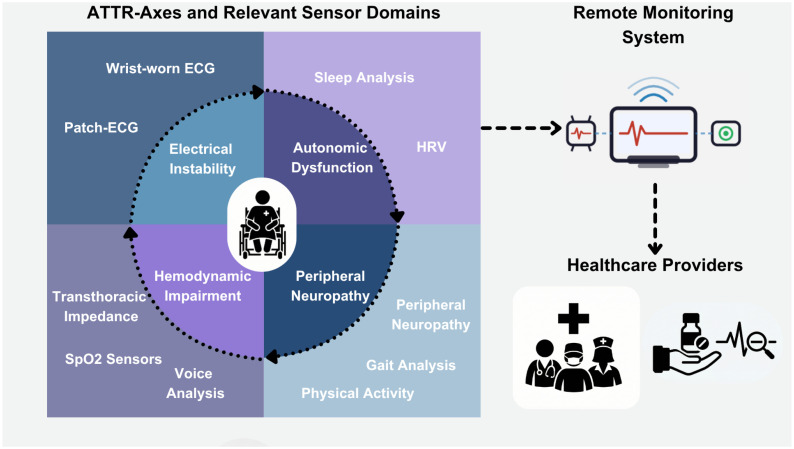
Graphical abstract depicting the proposed ATTR-axes and their association with sensor domains.

In ATTR, four interconnected pathophysiological axes are particularly well suited for longitudinal digital phenotyping: electrical instability and conduction disturbances, autonomic dysfunction, hemodynamic impairments and fluid dynamics, and peripheral neuropathy and motor dysfunction [8]. These axes interact dynamically with each other: electrical instability can exacerbate hemodynamic impairments, autonomic dysregulation can modulate rhythm patterns, and neuropathy can alter activity metrics that influence cardiac interpretation. Consequently, ATTR provides a compelling framework for multimodal sensor fusion rather than isolated parameter monitoring (Figure 1).

This review summarizes current findings on wearable sensor technologies in ATTR amyloidosis, defines potential digital biomarkers at the signal and feature level, and proposes a disease-specific conceptual framework for integrating multimodal data from wearable devices into longitudinal ATTR management. By positioning ATTR as a multisystem model for digital phenotyping, we aim to outline both the opportunities and methodological requirements necessary to advance wearable device monitoring from feasibility to clinically actionable disease surveillance.

This is a narrative review and does not follow a systematic review methodology. Relevant literature was identified through PubMed searches combining the terms [‘ATTR amyloidosis’, ‘transthyretin’, ‘cardiac amyloidosis’] with [‘wearable’, ‘sensor’, ‘telemonitoring’, ‘digital biomarker’, ‘accelerometer’, ‘photoplethysmography’, ‘ECG patch’], supplemented by hand-searching of reference lists of key articles and recent expert consensus documents. No formal inclusion/exclusion criteria were applied, and studies were selected based on their conceptual relevance to ATTR-specific monitoring domains and to illustrate methodological templates from adjacent cardiovascular and neurological cohorts. Therefore, the clinical recommendations and biomarker framework presented here reflect the authors’ synthesis rather than a pre-specified evidence hierarchy.

## 2. Categories and Technology of Wearable Devices

Wearable monitoring platforms can be categorized according to their primary sensing modality and the physiological signal they capture. In the context of ATTR, three sensor classes are particularly relevant: electrocardiographic monitors, photoplethysmography sensors, and inertial measurement units (IMU). The following paragraphs present a brief introduction to existing wearable device systems, which are more sophisticated.

### 2.1. Electrocardiographic Monitoring Devices

Wearable ECG systems record cardiac electrical activity using single-lead or multi-lead configurations and have demonstrated high diagnostic accuracy for arrhythmia detection, particularly atrial fibrillation (AF) [5,16,17,18,19]. These systems can be broadly divided into consumer-grade wrist-worn devices and medical-grade patch-based monitors.

Wrist-worn devices incorporate dry electrodes into the device casing and typically record short-duration single-lead ECGs (e.g., 30 s) upon user activation. The Apple Watch (Apple, Cupertino, CA, USA) was among the first consumer devices to receive regulatory clearance (FDA and CE marking) for AF detection using a single-lead ECG configuration sampling at 512 Hz [20]. Similar capabilities have since been integrated into other smartwatch platforms. These systems are optimized for intermittent rhythm assessment and event-triggered recording [17].

Patch-based ECG monitors are placed on the left upper thorax and provide continuous rhythm surveillance over extended periods, typically up to 14 days. The Zio Patch (iRhythm Technologies, San Francisco, CA, USA), an FDA-cleared adhesive single-lead ECG device that samples at 200 Hz with a bandwidth of 0.67–40 Hz, demonstrated superior arrhythmia detection compared with conventional 24-h Holter monitoring, largely attributable to prolonged recording duration [19]. Continuous ECG patches enable extraction of time-series features beyond binary arrhythmia detection, including AF burden, heart rate variability (HRV), ectopy frequency, and conduction trend analysis.

Smartphone-connected electrode systems, such as KardiaMobile (AliveCor, Mountain View, CA, USA), use handheld electrodes to acquire single-lead ECGs on demand. This device records a 30-s single-lead ECG with a sampling rate e of 300 Hz while using a filter of 0.05–40 Hz. Clinical trials have demonstrated their utility in AF screening and increased likelihood of capturing transient arrhythmias during symptomatic episodes [18,21,22]. For ATTR populations, prolonged rhythm monitoring is particularly relevant given the high prevalence of atrial fibrillation, cardiac arrhythmia necessitating pacemaker implantation, and progressive conduction disease [15].

Accurate feature extraction from ECG data (e.g., HRV) relies on adequate temporal resolution, which is achieved by sampling rates of 1000 Hz in stationary, medical-grade ECG devices. However, this leads to significant data and energy consumption, which exceeds capacities for mobile applications, because of which most wearable ECG devices apply a sampling rate between 200 and 512 Hz [23]. Downstream interpolation enables reasonably accurate feature extraction, such as automated R-peak detection and R-R interval measurement. Capturing ECG data during movement introduces significant noise. Because of this, before any downstream feature is computed, wearable ECG is typically band-pass filtered (commonly 0.05–40 Hz Butterworth filter) to suppress baseline wander, with a 50 or 60 Hz notch for power-line interference. A broader bandwidth and wavelet denoising is used when QRS morphology must be preserved rather than heart rate [24]. Signal quality is then assessed segment-wise using complementary indices that together cover different failure modes: kurtosis and skewness of the amplitude distribution flag flat-line or saturated segments, beat-detector agreement (bSQI) between two independent R-peak detectors identifies missed or spurious beats, and template-matching cross-correlates each detected beat against a segment-wise averaged beat [25]. None of these SQI thresholds have been calibrated in ATTR cohorts, where low voltage, frequent ectopy, and conduction disease are expected to reduce bSQI performance and template stability.

### 2.2. Photoplethysmographic Sensors

Photoplethysmography is a non-invasive optical sensing technique that estimates cardiovascular dynamics by detecting changes in light absorption related to pulsatile blood flow [26,27,28]. An LED emits light into the tissue, and a photodetector measures variations in reflected or transmitted light intensity corresponding to the cardiac cycle. The choice of LED is determined by its emitted wavelength, with blood oxygen saturation typically requiring both infrared (~850 nm) and red wavelength (~660 nm) emitting diodes [29]. Broadly, PPG can be classified into either transmissive, with the photodetector located on the opposite side of the LED and skin tissue in between both, or reflective type, with the LED and photodetector being located next to each other on top of a section of skin tissue. These sensors sample at 21–64 Hz to enable downstream feature extraction while handling battery and storage expenditure [30]. Recorded raw data are typically band-pass filtered between 0.5 and 15 Hz to retain the fundamental and first harmonics of the pulse while removing baseline wander from respiration and vasomotion [31,32]. Quality assessment operates at the pulse rather than beat level. The most widely adopted indices are a skewness-based SQI, the perfusion index (ratio of the AC to DC component of the PPG, reflecting signal strength), and pulse-template correlation analogous to the ECG approach [28,31]. For pulse-rate-variability analysis specifically, segment rejection is preferred over waveform reconstruction, since reconstruction distorts inter-beat intervals in ways that can mimic or mask autonomic signal. The provided mechanisms discussed above degrade the perfusion index and template-correlation scores simultaneously, and ATTR-specific SQI thresholds are likely to be required rather than the defaults carried over from healthy-cohort validation. Such PPG sensors are widely integrated into wrist-worn devices and enable passive, continuous acquisition of heart rate, pulse rate variability, peripheral oxygen saturation (SpO_2_), respiratory rate (derived), and sleep-related physiological patterns.

Unlike ECG devices, PPG operates without user activation, allowing uninterrupted background monitoring. Advanced signal processing enables extraction of pulse morphology features, inter-beat intervals, and pulse transit dynamics. Models for deriving these metrics from the sensor readings are the subject of ongoing research and are being expanded into new readings such as blood pressure [32]. However, PPG-derived metrics are susceptible to motion artifacts, low peripheral perfusion, and irregular rhythm disturbances.

Despite these limitations, the unobtrusive and passive nature of PPG makes it attractive for long-term monitoring in older populations, including patients with ATTR amyloidosis.

### 2.3. Activity Trackers and Inertial Measurement Units

Activity monitoring devices typically rely on force sensors capable of measuring linear or angular motion, called accelerometers or gyroscopes, respectively. Briefly, these sensors measure the physical displacement of a proof mass attached to a suspension system compared with a reference frame [33,34,35]. Optimal sampling rates of accelerometers and gyroscopes differ according to the subjects’ velocity (i.e., 100 Hz for walking vs. 200 Hz for running) as well as sensor placement and desired feature extraction [36,37,38]. Sensors placed at the hip sampling at 50 Hz provided the most accurate data to distinguish between activities of daily living [36]. For clinical analysis, raw acceleration vectors measured in m/s^2^ are processed using algorithmic models to extract features such as step count, distance traveled, energy expenditure, sedentary time, and possible fall detection.

Research-grade devices such as the ActiGraph platform (Ametris, Pensacola, CA, USA) sample at 30–100 Hz have been widely used in cardiovascular and neurological studies [3,39]. However, consumer-grade accelerometer-based wearables increasingly demonstrate sufficient signal fidelity for longitudinal monitoring applications. More sophisticated signal processing modalities allow for a detailed segmentation of body position and movement information, with consistently lower computational cost, allowing their implementation in wearable devices [40].

Beyond general activity tracking, wearable inertial measurement units (IMU) incorporate accelerometers, gyroscopes, and magnetometers to enable detailed and quantifiable gait analysis [2]. Activity measurement systems comprise 2–8 wearable IMU sensors and are commonly placed at the lower limbs, waist, or lumbar (L4) region to capture temporal and spatial gait parameters [2,41]. Extractable features include stride length, cadence, gait variability, step asymmetry, turning stability, and postural sway. Commercial systems such as LEGSys (BioSensics, Newton, MA, USA) with sampling rates of 100 Hz have demonstrated feasibility in differentiating neurological gait phenotypes in real-world settings [42,43]. For ATTRv amyloidosis, where progressive sensorimotor polyneuropathy is common, IMU-derived gait variability and asymmetry indices represent particularly promising candidate digital biomarkers for at-home disease monitoring.

To contextualize the technical characteristics of the main wearable sensor modalities relevant for ATTR monitoring, Table 1 summarizes the signal characteristics and disease-specific issues of ECG-, PPG-, and IMU-based systems. This comparison shows that each modality captures different physiological dimensions and has different susceptibility to artifacts and disease-related signal distortions.

## 3. Evidence of Wearables in ATTR-Amyloidosis

The evidence for monitoring ATTR amyloidosis using wearables remains sparse and heterogeneous, with most data coming from small observational cohorts or sub-studies embedded in clinical trials. Existing data can be grouped into three domains: early detection, rhythm monitoring, and functional assessment. A broader evidence base from heart failure and neurological populations provides biological plausibility and methodological templates but does not replace ATTR-specific validation.

### 3.1. Early Diagnosis of ATTR-CM

Despite increasing awareness and improved diagnostic pathways, cardiac amyloidosis remains substantially underdiagnosed. Artificial intelligence (AI) models trained on 12-lead ECG and echocardiographic data have demonstrated promising performance in detecting cardiac amyloidosis in tertiary cohorts [44,45,46]. However, these approaches depend on clinical acquisition infrastructure and specialist interpretation.

To expand screening accessibility, Sangha et al. evaluated an AI algorithm trained on single-lead wearable ECG data to identify ATTR-CM [47]. Using noisy real-world wearable recordings, the model achieved a sensitivity of 85% and specificity of 80% (area under the curve (AUC) 0.90, 95% CI 0.88–0.92). Although preliminary and derived from selected cohorts, this study demonstrated that diagnostic signal features characteristic of ATTR-CM may be extractable even from wearable-derived, real-world ECG data. Additionally, the single-center retrospective Willem study recently presented promising results of recognizing ATTR-CM from clinical 12-lead ECG with an AUC of 0.88 (95% CI 0.85–0.91) [48]. Although only stationary ECG devices were used in this trial, the Willem AI was trained on different ECG data sources, including wearables, which might enable portable ECG-based identification of ATTR-CM in the future.

However, implementation in screening programs requires external validation in populations with varying disease prevalence, as the positive predictive value and associated costs are highly dependent on prevalence. In addition, the performance of the model must be evaluated under real-world signal variations, including motion artifacts and arrhythmic disturbances. At present, portable ATTR screening should be considered hypothesis-generating rather than clinically established.

### 3.2. Arrhythmia Detection in ATTR-Amyloidosis

Atrial fibrillation and conduction abnormalities are highly prevalent in ATTR-CM and are associated with increased morbidity and hospitalization risk [15,49,50]. Non-sustained ventricular tachycardia also occurs frequently and may contribute to sudden cardiac death risk [51]. Current expert consensus recommends ambulatory rhythm monitoring, with prolonged monitoring considered in selected patients [52].

Wearable ECG systems offer extended monitoring durations compared with conventional 24-h Holter recordings. In a retrospective study of 38 ATTR-CM patients undergoing 14-day patch-based ECG monitoring, AF was detected in 21.3% and non-sustained ventricular tachycardia in 81.6% of patients [16]. Detection rates exceeded those observed in a stroke-evaluation control cohort, highlighting the high arrhythmic burden in ATTR-CM.

Although limited by small sample size and retrospective design, this study supports the feasibility of long-term wearable rhythm surveillance in ATTR-CM. Similarly, implantable loop recorder case series have demonstrated a high frequency of actionable arrhythmic events over extended follow-up [53], underscoring the clinical relevance of continuous rhythm assessment. Evidence from non-ATTR populations further supports prolonged wearable monitoring. In comparative studies, extended patch monitoring detected significantly more arrhythmic events than short-term Holter recordings, largely due to longer recording duration [19,54]. Large-scale smartwatch studies have further demonstrated scalable AF detection in general populations [5].

Whether these detection rates translate into ATTR-CM and lead to improved outcomes remains to be proven. Importantly, beyond binary arrhythmia detection, the wearable ECG enables quantification of AF burden and longitudinal RR variability trends, which may be more meaningful in progressive infiltrative cardiomyopathies.

### 3.3. Wearable-Based Functional Assessment

Functional limitation is central in ATTR-CM and typically assessed with New York Heart Association (NYHA) class and the 6-min walk test (6MWT) [55,56,57]. However, episodic testing may fail to capture day-to-day variability or gradual decline.

A substudy within ATTRibute-CM investigating the use of a wearable Opal V2C System (APDM Wearable Technologies, Portland, OR, USA) to conduct a 6MWT demonstrated high concordance between wearable-derived and conventionally measured 6MWT distance (r = 0.998, 95% CI 0.992–0.999) [58]. However, measurements were obtained under controlled clinical conditions, thus performance and adherence in unsupervised at-home settings remain to be established. Given the relevance of 6MWT as a trial endpoint [59], home-based assessment could become attractive if robustness to real-world variability is demonstrated.

### 3.4. Neuropathy and Gait Monitoring in ATTRv

Hereditary ATTR often manifests as progressive sensorimotor polyneuropathy (PNP) [12]. Traditional assessment relies on clinical scales and neurophysiological tests, which are episodic and examiner-dependent. To date, no ATTR-specific evidence exists on the use of wearables to monitor progression or treatment-related remission of neuropathy. Nonetheless, small studies in diabetic neuropathy, which presents overlapping features with ATTR-PNP, exhibited the feasibility and usefulness of wearable vibration or electrical stimulation systems to improve plantar sensation and motor function [60,61]. Another interesting yet explorative research field, with potential transferability to ATTR-PNP, resembles the application of pedobarography enabled by in-sole pressure and temperature sensors to identify early deterioration in plantar sensation [62,63].

Three-dimensional camera-based gait analyses have been able to distinguish asymptomatic carriers from symptomatic ATTRv patients and to detect longitudinal changes in specific gait parameters such as toe-off time and pelvic rotation, which were undetected by traditional visual observation [13,64]. However, these systems require a sophisticated and expensive laboratory infrastructure. Wearable IMU offer the possibility of quantifying gait variability, asymmetry, and falls at home. Although ATTR-specific studies on wearable devices are lacking, neurological populations such as Parkinson’s disease and multiple sclerosis patients have demonstrated the feasibility and correlation with clinical motor scales [2,65,66,67,68]. Fall detection is another relevant application. Automated fall detection systems are highly sensitive but can suffer from a high false positive rate [69]. In ATTR, falls can be caused by both neuropathy and conduction-related syncope, suggesting that multimodal integration (portable gait pattern analysis and ECG) could improve etiological differentiation.

The TelePD study will be the first to examine the feasibility of wearable socks for measuring gait and balance data at home, as well as the response to balanced telerehabilitation compared with unsupervised exercises [70]. This study will provide valuable insights into the use of wearables during remote treatment and their reliability as a tool for physicians to track therapy success.

### 3.5. Exploratory Digital Biomarkers

Wearable-derived HRV and sleep metrics may reflect autonomic dysfunction, and studies have demonstrated predictive value of HRV measurements for sudden cardiac death [71]. However, in ATTR interpretation can be confounded by atrial fibrillation, ectopy, and conduction disease, necessitating further research including ATTR populations. In HF, early detection of deteriorating heart function is essential to prevent hospitalization or further adverse outcomes. Remote monitoring approaches that monitor fluid changes by thoracic impedance systems or voice biomarkers captured with a smartphone have shown promise in broader HF cohorts [6,72], but remain unvalidated in ATTR.

## 4. Why Extrapolation from Adjacent Diseases Is Plausible—But Requires Validation

ATTR progresses slowly with incremental deterioration often preceded by subtle changes in arrhythmic burden, resting heart rate, activity tolerance, autonomic regulation, and motor stability (mostly in ATTRv). These processes evolve longitudinally and may be detectable through continuous physiological sampling in at-risk cohorts rather than episodic clinical encounters. The availability of disease-modifying therapies increases the need for dynamic monitoring of progression and therapeutic response. Wearables offer biologically coherent measurement domains: validated AF detection is directly actionable in ATTR-CM [5,16,73], may potentially detect subtle fluid status changes prior to HF decompensation [6], and accelerometer-based physical activity or gait metrics align with known disease trajectories (Table 2). Evidence supporting the clinical value of these domains, albeit to varying degrees, has been established across a number of pathophysiologically adjacent disease states (Table 3).

The clinical findings discussed above are not unique to ATTR amyloidosis but can also be present in AL amyloidosis, an infiltrative disease from clonal plasma cell dyscrasia in which monoclonal immunoglobulin light chains deposit in the myocardium and, in a subset of patients, in peripheral and autonomic nerves. In both AL and ATTR amyloidosis, polyneuropathy-related gait instability and fall risk, orthostatic hypotension, and atrial fibrillation are documented. Nonetheless, from our point of view, several features position ATTR amyloidosis as the more informative setting for wearable-based clinical monitoring than AL. First, polyneuropathy-related gait instability and fall risk are cardinal in ATTRv and increasingly recognized in ATTRwt, whereas peripheral nerve involvement affects only 10–35% of AL patients and rarely dominates the functional picture, given the more dominant hematologic and cardiac clinical phenotype. Second, atrial fibrillation differs quantitatively between both entities: prevalence reaches ~80% in ATTRwt versus 28–33% in AL cardiac amyloidosis, with an approximately ten-fold higher adjusted risk in ATTR and a stage-dependent rise to 94% in stage III disease, which is not observed in AL [49,76]. Continuous rhythm surveillance therefore yields substantially more actionable data in ATTR. Third, ATTR cardiomyopathy lacks a circulating biomarker analogous to dFLC in AL. Consequently, functional, hemodynamic, and rhythm-based endpoints delivered by wearable-derived digital biomarkers might provide a greater benefit for clinical monitoring and treatment response. This gap becomes even more important given the emergence of disease-modifying therapies for ATTR cardiomyopathy.

### 4.1. ATTR-Specific Signal Distortions and Implications for Validation

However, extrapolation from HF or neurological cohorts assumes that signal-disease relationships remain stable in the context of infiltrative cardiomyopathy, conduction disease, and peripheral neuropathy. ATTR-specific pathophysiology, such as autonomic and small-fiber neuropathy and arrhythmic heart rate, may distort conventional digital biomarkers. For example, PPG signal quality relies heavily on physiological tissue perfusion, which is influenced both by high arrhythmic burden and peripheral vasoconstriction in ATTR amyloidosis. Disturbing the expected PPG waveform morphology by flattening of pulse amplitude and the dicrotic notch reduces signal-to-noise ratio and therefore might prevent the longitudinal use of PPG data for monitoring ATTR. However, extrapolating from a pilot study in patients with severe aortic stenosis undergoing transcatheter valve replacement, PPG analysis demonstrated discriminatory value to classify patients along intracardiac pressure measurements [77]. Although the study did not investigate longitudinal PPG data, this demonstrates the feasibility of using PPG signals in diseases with perfusion disturbances.

Clinical ECGs of ATTR patients, on the other hand, are notoriously different to those of healthy persons. Conduction disease (e.g., delayed AV-conduction and bundle branch blocks), high atrial fibrillation burden, and low voltage phenomena are frequent in ATTR-CM and might therefore further interact with already noisy wearable-derived ECG data. This can lead to impaired feature extraction, such as R-peak detection and template-matching, and thus decrease the signal quality. Several wearable ECG devices, such as those discussed above, demonstrated the feasibility of data analysis based on ECGs polluted with motion artifacts. Additionally, in a pilot study, symmetric projection attractor reconstruction derived from ECG in ATTR patients proved feasible and was able to distinguish stable patients from those with deteriorating heart function [78].

While these results are promising, the utility of wearable-derived data in ATTR patients for advanced analysis and remote longitudinal monitoring has yet to be validated. Until prospective studies demonstrate reproducible associations with clinically adjudicated endpoints, the discussed results must be regarded as biologically plausible but unvalidated.

### 4.2. ATTR-Specific Clinical Use Case

Cardiac decompensation remains frequent in ATTR-CM despite disease-modifying therapy, with about 25% of patients on transthyretin stabilizing treatment (e.g., tafamidis or acoramidis) presenting with cardiovascular hospitalizations, mostly due to heart failure decompensation, in contemporary trial cohorts [75,79]. Outpatient detection of subclinical congestion before symptomatic decompensation is therefore an unmet need that wearable monitoring is well-suited to address. In specialized ATTR centers, patients could be equipped with smartwatches providing continuous PPG and wearable single-lead ECG devices, with an optional wrist- or chest-mounted IMU for activity capture. In this scenario, usable longitudinal data are of key importance; as a result, patients would need to be instructed on wear-time expectations, charging routines, and secure data transfer. Digital biomarkers such as resting and nocturnal heart rate, daily step count and active minutes, sleep continuity, and rhythm-detection alerts are securely streamed to the center, where they are periodically reviewed. Additionally, IMU-derived measures of gait cadence and postural stability may be referenced and compared against each patient’s individual baseline. Such telemonitoring data can be integrated into the routine assessment of patients’ cardiac and neurologic status and complement clinical scores such as the Peripheral Neuropathy Disability (PND) score.

**Figure 2:**
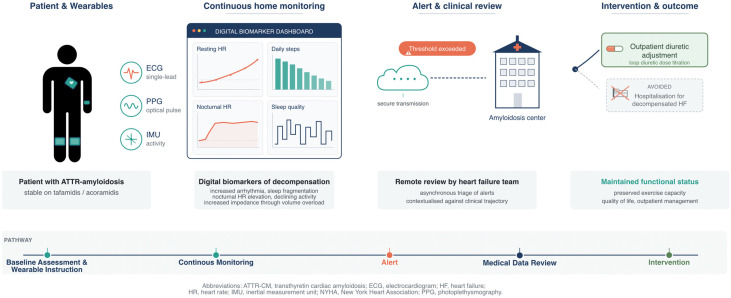
Clinical integration pathway for wearable-based monitoring in ATTR amyloidosis.

Plausible and concurrent drifts across several biomarkers in between clinical visits would prompt outreach to the patient and, where indicated, outpatient diuretic titration or a clinic visit. The premise of this pathway is that wearable-derived warning signs precede patient-perceived symptoms by days to weeks, which is the window in which adjustment of oral therapy can plausibly prevent admission. Figure 2 depicts this monitoring pathway using wearables in ATTR amyloidosis. To date, this continuous monitoring and intervention approach has not been tested in ATTR amyloidosis and may be blunted by disease-specific signal distortions as discussed above. However, we believe that ATTR amyloidosis presents a model disease for telemonitoring due to the high prevalence of arrhythmic and heart failure events, autonomic dysfunction, and the slow progressive nature of this disease. Actionable signals and cut-offs based on wearable-derived digital biomarkers will need to be validated in prospective validation studies but may ultimately benefit patients’ disease trajectory.

### 4.3. Implementation Barriers and Future Directions

Beyond scientific validation, there are significant structural barriers to implementation in clinical practice. While most physicians believe in a future role of wearables and telemonitoring applications in health care, a significant proportion also experience reimbursement issues, high investment cost, and insufficient technical equipment or experienced personnel. In a German web-based survey study among outpatient cardiologists, internists, and practitioners, about 46% reported a high degree of insufficient system interoperability [80].

The latter might stem from the lack of standardized technical properties of wearable platforms, such as differences in sampling frequency (e.g., patch-based ECG typically at 200–512 Hz versus consumer wrist PPG at 25–100 Hz), preprocessing pipelines, and proprietary feature-extraction algorithms. For a single underlying physiological metric (i.e., resting heart rate), the value reported by a Fitbit, an Apple Watch, or a medical-grade ECG patch may differ in ambulatory conditions, even more in the presence of AF [81,82]. The lack of industry-wide standards not only limits reproducibility across studies but also complicates the real-world usage of varying wearable systems for telemonitoring.

Interoperability with electronic health records remains limited. Routine integration of wearable-derived data into clinical workflows requires that sensor output be translatable into structured electronic health record entries. HL7 FHIR “Observation” resources, combined with LOINC codes (e.g., 8867-4 for heart rate) and UCUM units, have emerged as the prevailing standard for this translation. However, most consumer-grade devices do not provide API interfaces for clinical integration. Therefore, specialized disease centers might require either device-agnostic middleware or partnerships with manufacturers willing to provide clinical-grade data streams.

Regulatory classification of wearables further varies markedly by jurisdiction. In the European Union, devices intended for diagnosis, monitoring, or treatment fall under the Medical Device Regulation (EU 2017/745) and are classified I, IIa, IIb, or III depending on risk [83]. Most diagnostic wearables for arrhythmia detection are IIa. In the United States, the FDA uses a Class I–III framework with 510(k), De Novo, or PMA pathways, and in 2024–2026 issued revised guidance on Clinical Decision Support software and Software as a Medical Device.

Germany’s digital health applications (Digitale Gesundheitsanwendungen, DiGA) pathway, administered by the BfArM, provides a structured reimbursement route for digital health applications classified as MDR Class I/IIa (and, since 2024, IIb), requiring CE marking, demonstrating a positive care effect, and adherence to data-protection and interoperability standards. Comparable pathways have since been adopted or announced in France (PECAN) and Belgium (mHealthBelgium). No such pathway currently exists specifically for wearable-based rare-cardiomyopathy monitoring, which represents a significant barrier to the translation of the monitoring concepts discussed in this review.

Importantly, the demographic profile of ATTRwt amyloidosis presents an additional challenge for implementation. Patients tend to be older and may have lower digital literacy, sensory limitations, or lower adherence to device-based self-management strategies. Device usability, passive data collection, minimal user interaction requirements, and caregiver-supported workflows are therefore critical for sustainable adoption in this population.

Future research should combine prospective, phenotype-stratified validations with implementation science approaches anchored in specialized amyloidosis centers. Multimodal sensor fusion, predefined triage algorithms, and cost-benefit analyses will be essential to transition wearable monitoring from feasibility to clinically meaningful integration into ATTR care. Finally, the use of wearables could enable centers with interdisciplinary ATTR expertise to incorporate scalable remote monitoring for a widespread provision of medical care.

## 5. Conclusions

Wearable sensor technologies offer a biologically coherent framework for early detection and long-term monitoring in transthyretin amyloidosis. The progressive and multisystemic nature of ATTR aligns with domains that can be measured with ECG, PPG, and inertial motion sensors, and early studies demonstrate the feasibility of rhythm and function assessment. However, ATTR-specific validation remains limited, and prospective studies linking wearable-derived features to adjudicated clinical outcomes are needed before routine implementation can be recommended. Furthermore, successful integration must consider the demographics of ATTRwt, where advanced age and limited digital literacy may constrain the use of technology-dependent monitoring strategies.

Wearables should therefore be viewed as a promising but still experimental adjunct to structured multidisciplinary care. Importantly, wearables are intended to complement, rather than substitute, the established clinical methodologies that underpin current ATTR management. Their ultimate value depends not only on the accuracy of the signals but also on validated clinical impact and practical applicability in the patient populations for which they are intended.

## Figures and Tables

**Table 1:** Technical Comparison of Major Wearable Sensor Modalities Relevant to ATTR Monitoring.

Parameter	Electrocardiography	Photoplethysmography	Accelerometry/Gyroscope
**Primary Measured Signal**	Cardiac electrical potential difference	Optical pulse waveform (blood volume changes)	Linear acceleration and angular velocity
**Sensor Placement**	Chest Strap, Chest Patch	Wrist or arm band	Wrist, chest, back, hip, ankles
**Signal Sampling Rate**	200 Hz–512 Hz	21 Hz–100 Hz	20 Hz–400 Hz
**Signal Domain**	Bioelectrical	Optical/Hemodynamic	Mechanical/Kinematic
**Core Extractable Features**	RR intervals, PR/QRS duration, AF burden, HRV	Pulse rate variability, SpO_2_, pulse morphology, sleep-related metrics	Step count, stride length, gait variability, asymmetry index, fall detection
**Strengths**	Gold standard for arrhythmia detection; direct electrical measurement	Passive continuous monitoring; integrated in consumer devices	Objective functional assessment; sensitive to motor decline
**Key Limitations**	Limited leads in wearables; motion artifacts; electrode–skin dependency	Motion artifacts; low perfusion sensitivity; AF reduces accuracy	Algorithm-dependent feature derivation; placement-sensitive
**ATTR-Relevant Applications**	AF burden tracking, conduction trend monitoring, autonomic HRV analysis	Resting HR trends, autonomic modulation	Neuropathy progression, activity decline, fall risk
**Continuous Monitoring Capability**	Intermittent active (wrist-worn device) or continuous passive (patch)	Continuous passive	Continuous passive

Legend: HRV = Heart rate variability, SpO_2_ = peripheral oxygen saturation, AF = atrial fibrillation, ATTR = transthyretin amyloidosis, HR = Heart rate.

**Table 2:** Wearable and digital biosensor evidence specifically generated in ATTR amyloidosis cohorts.

Study (Year)	Journal	Design	Modality/Device	Endpoint	Key Finding	Evidence Level *
Sangha et al. (2024) [47]	*European Heart Journal*	Retrospective case-control AI algorithm validation study	Single-lead I derived from 12-lead ECG, noise-augmented for wearable simulation	(Early) Detection of ATTR-CM with the AI-ECG model	AUC to detect ATTR-CM was 0.90 (95% CI: 0.88–0.92), sensitivity and specificity was 0.85 and 0.80, respectively	Low (conference abstract; retrospective)
Soman et al. (2025) [58]	*Future Cardiology*	Cross-sectional pilot within phase 3 randomized controlled trials	Foot- and lumbar-mounted IMU sensors (concurrent in-clinic 6MWT)	Concordance of wearable-derived 6MWD vs. observer-measured 6MWD	Mean 6MWD 330.3 m (conventional) vs. 335.1 m (wearable); mean difference 4.7 m (SD 10.95) Pearson *r* = 0.998 (95% CI 0.992–0.999); concordance correlation coefficient = 0.997 (95% CI 0.991–0.999)	Low (small pilot; in-clinic, not at-home)
Vilas-Boas et al. (2020) [13]	*Frontiers in Neurology*	Cross-sectional case-control	3D vision system (camera-based gait analysis); not body worn	Distinction of disease stages by gait parameters in ATTR	Successful distinction between healthy subjects, asymptomatic carriers of variant mutations and symptomatic patients with gait parameters such as stride duration, stride length, step duration, step length, stance duration and swing duration (*p ≤ 0.001*)	Low (camera-based, not ambulatory wearable)
Bruce et al. (2024) [16]	*Heart Rhythm O_2_*	Single-center retrospective case-control	2-week non-invasive ambulatory adhesive ECG patch	Prevalence of atrial fibrillation/flutter and NSVT	14-day patch monitoring detected Atrial fibrillation/flutter in 26.3% and NSVT in 81.6% of ATTR-CM patients	Low (single-center, retrospective, modest sample size)
Sehrawat et al. (2025) [51]	*Journal of Cardiovascular Electrophysiology*	Single-center retrospective cohort	Clinically indicated ambulatory Holter monitoring (body-worn, clinical-grade)	Primary: incident sustained VA Secondary: all-cause mortality	Sustained VA occurred in 11 patients (5.1%; incidence 1.8 per 100 person-years, all monomorphic VT); 46 patients (21%) died	Low–Moderate (largest ATTR-specific ambulatory-monitoring cohort, but retrospective and single-center)
Vilas-Boas et al. (2022) [64]	*Journal of Clinical Medicine*	Longitudinal case series	3D vision system (camera-based gait analysis); not body worn	Identify gait abnormalities and progression over 18 months in ATTR patients	Identified toe-off delay, excessive pelvic rotation, hip extension, external transverse rotation and knee flexion as qualitative gait abnormalities in ATTR patients	Very low (case series, single center, camera based and not wearable)
Abbasi et al. (2024) ^+^ [53]	*JACC: Cardio Oncology*	single-arm pilot	Implantable loop recorder	Burden of clinically actionable conduction-system disease and arrhythmias in ATTRwt-CM	10 of 24 patients (42%) had arrhythmic or conduction-system events: AF in 9 (7 symptomatic, 2 new-onset asymptomatic), ventricular arrhythmia in 1 (exertional syncope) 3 patients required dual-chamber permanent pacemakers for symptomatic conduction disease (2 AF with slow ventricular response, 1 Mobitz II second-degree AV block) 1 additional patient with previously undocumented complete heart block received a CRT-pacemaker; 1 received an ICD	Low (uncontrolled single-arm pilot; implantable rather than wearable)

Legend: ATTR = transthyretin amyloidosis, CM = cardiomyopathy, ECG = electrocardiography, AF = atrial fibrillation, 6MWD = six-minute walking distance, VA = ventricular arrhythmia, NSVT = non-sustained ventricular tachycardia, CI = confidence interval, AUC = area under the curve, AV = atrio-ventricular, CRT = cardiac resynchronization therapy, ICD = implantable cardiac defibrillator. * GRADE-inspired qualitative grading: high (large prospective trial or multiple consistent studies); moderate (single robust prospective study or several consistent observational ones); low (small observational, pilot, or retrospective); very low (case series, *n* < 10). ^+^ Implantable, not wearable; included here because this is the longitudinal ATTR-specific cardiac-rhythm monitoring data and serve as biological-plausibility anchors for wearable rhythm surveillance.

**Table 3:** Extrapolated wearable-monitoring evidence from adjacent disease cohorts, with assessment of transferability to ATTR amyloidosis.

Monitoring Domain	Adjacent Cohort	Representative Studies	Modality	Key Finding	ATTR Transferability	Confidence
**AF detection**	General population; ED palpitations; community screening	Apple Heart Study, *n* = 419,297 (Perez 2019) [5] Smartwatch AF accuracy meta-evidence [17]; Apple Watch ECG diagnostic accuracy [20] Smartphone-based event recorder RCT [21]; iPhone ECG community screening [22] 14-day adhesive patch vs. 24-h Holter [19]; wearable ECG clinical review [18]	Wrist PPG with irregular-rhythm notification + ECG patch confirmation Smartwatch single-lead ECG Adhesive long-wear patch	0.5% of participants notified; 84% PPV among notified for confirmed AF (Apple Heart Study) Smartwatch ECG and PPG show high sensitivity and specificity for AF in pooled analyses Extended-wear patches detect 2–3× more AF than 24-h Holter	High: AF prevalence in ATTR-CM substantially exceeds the general population, so detection algorithms validated at low prevalence transfer favorably to higher-prevalence settings	**Moderate**
**HF decompensation monitoring**	Chronic heart failure	Multiparametric wearable sensor for HF rehospitalization, open-label concurrent-control trial [72] Telemonitoring in HF, meta-analysis [7] Voice analysis for early fluid overload—VAMP-HF [6] Lifestyle digital intervention in CVD [74]	Multiparametric chest patch (HR, activity, respiration, posture) Smartphone-based voice biomarker App-based lifestyle and self-monitoring tools	Wearable sensor-guided strategy reduced HF rehospitalization versus standard care (Boehmer) Telemonitoring meta-analysis showed reductions in mortality and HF rehospitalization Voice biomarkers detect fluid overload preclinically and correlate with congestion status	Moderate: restrictive physiology, fixed stroke volume, and high arrhythmic burden in ATTR-CM may attenuate the early-warning signature relative to non-amyloid HF cohorts ATTR-specific thresholds and validation in disease-modifying-therapy populations are required	**Low to Moderate**
**Functional capacity (6MWT and step count)**	Heart failure and cardiomyopathy	Wearable physical activity monitors in CVD, systematic review *n* = 11,464 [3] Validation of consumer wrist-worn activity trackers [34] Natriuretic peptides, 6MWT and QoL as clinically meaningful HF endpoints [56] Disease-specific anchor: 6MWT prognostic value in ATTR-CM [14]	Wrist accelerometer/smartwatch Lumbar and foot-mounted IMU Smartphone app step count	Wearable-derived step count and ambulatory 6MWD correlate with observer-measured functional capacity Activity-monitor metrics independently prognosticate hospitalization and mortality in CV cohorts 6MWT is an established surrogate endpoint in HF trials	High: 6MWT is already an accepted clinical-trial surrogate in ATTR-CM [55,56,57,58,59,60,61,62,63,64,65,66,67,68,69,70,71,72,73,74,75] and predicts mortality at the National Amyloidosis Centre cohort level [14]	**Moderate to High**
**Heart-rate variability and autonomic function**	Chronic HF; diabetic and other autonomic neuropathy	Short-term HRV strongly predicts SCD in chronic HF [71]	ECG-derived short-term HRV (time- and frequency-domain)	Reduced short-term HRV strongly predicts sudden cardiac death in chronic HF, independent of LVEF and clinical class	Moderate: autonomic dysfunction is well documented in ATTRv and is increasingly recognized in ATTR-CM	**Low to Moderate**
**Gait, balance, and neuropathy monitoring**	Parkinson’s disease; progressive supranuclear palsy; diabetic peripheral neuropathy	Quantitative gait analysis with wearable sensors, comprehensive review [2] Wearable gait classification of fallers vs. non-fallers [41] Remote at-home wearable gait in PSP vs. PD [42] Single-sensor wearable gait in PD [65] Lower-limb kinematics with reduced IMU count [40] Time–frequency assessment of gait accelerometry [68] Plantar electrical stimulation in DPN [60]; focal muscle vibration in DPN [61] Smart insole pressure/temperature for neuropathic feet [62]; IoT foot neuropathy [63]	Body-worn IMU at lumbar, shank, or wrist Instrumented smart insoles (pressure/temperature) Smartphone inertial sensors	Cadence, stride-time variability, and postural sway track motor progression and predict falls Smart insoles detect neuropathic foot-pressure asymmetry and skin-temperature changes preclinically Wearable gait phenotypes discriminate parkinsonian syndromes	Moderate: ATTRv polyneuropathy is length-dependent and clinically resembles diabetic peripheral neuropathy, which supports biological transfer	**Moderate**
**Disease-progression tracking under disease-modifying therapy**	Multiple sclerosis; Parkinson’s disease	Smartphone-sensor digital outcome assessment in MS [67] Fall-detection methods in MS [69] Balance telerehabilitation and wearable technology in PD—TelePD [70] Wearable-tracked motor and mobility change in PD [66]	Smartphone IMU + active and passive tasks Wearable balance and gait sensors Patient-reported outcome integration	Continuous wearable-derived metrics detect treatment effect at smaller sample sizes than scheduled clinical scales Wearable balance and fall metrics provide complementary information to neurologist-administered scores	Moderate to high: with the proliferation of disease-modifying therapy in ATTR (tafamidis, acoramidis, vutrisiran, eplontersen), wearable endpoints offer the same statistical-power gain in principle	**Low to Moderate**

Legend: ED = early diagnosis, AF = atrial fibrillation, RCT = randomized controlled trial, ECG = electrocardiogram, PPG = photoplethysmography, ATTR = transthyretin amyloidosis, CM = cardiomyopathy, CVD = cardiovascular disease, HF = heart failure, HR = heart rate, IMU = inertial measurement unit, 6MWT = six-minute walk test, PD = Parkinson’s disease, MS = multiple sclerosis.

## Data Availability

No new data were created or analyzed in this study.

## Abbreviations

The following abbreviations are used in this manuscript:

6MWT	6-Minute Walk Test
AF	Atrial Fibrillation
AI	Artificial Intelligence
ATTR	Transthyretin Amyloidosis
ATTR-CM	Transthyretin Amyloidosis Cardiomyopathy
ATTR-PNP	Transthyretin Amyloidosis Polyneuropathy
ATTRv	Hereditary Transthyretin Amyloidosis (variant)
ATTRwt	Wild-Type Transthyretin Amyloidosis
AUC	Area Under the Curve
CE	Conformité Européenne
CI	Confidence Interval
CPE	Cardiopulmonary Exercise Testing
ECG	Electrocardiogram
FDA	Food and Drug Administration
HF	Heart Failure
HFpEF	Heart Failure with Preserved Ejection Fraction
HRV	Heart Rate Variability
IMU	Inertial Measurement Unit
LED	Light-Emitting Diode
MEMS	Micro-Electromechanical System
NYHA	New York Heart Association
PNP	Polyneuropathy
PPG	Photoplethysmography
SpO_2_	Peripheral Oxygen Saturation
TTR	Transthyretin
